# Primarily treated patients versus referred patients in the treatment of native septic arthritis of digits: a retrospective comparative study

**DOI:** 10.1186/s12891-020-03770-9

**Published:** 2020-11-27

**Authors:** Sang Ho Kwak, Jung Yun Bae, Youngkwang Oh, Hyo Seok Jang, Tae Young Ahn, Sang Hyun Lee

**Affiliations:** 1 Department of Orthopaedic Surgery, Pusan National University Yangsan Hospital, Yangsan, Republic of Korea; 2 Department of Orthopaedic Surgery, Inje University Haeundae Paik Hospital, Busan, Republic of Korea; 3 Department of Orthopaedic Surgery, Medical Research Institute, Pusan National University Hospital, Busan, Republic of Korea

**Keywords:** Septic arthritis, Methicillin-resistant *Staphylococcus aureus*, Empiric antibiotics, Surgical drainage, Finger injuries

## Abstract

**Background:**

Septic arthritis of digits needs urgent treatment. When treatments delayed or insufficient, patients may be referred to the upper-level hospital due to uncontrolled infection. We reviewed the treatment history of referred patients and compared the microorganisms and the clinical course of both primary and referred patients as relevant studies are rare.

**Methods:**

In this retrospective review of consecutive case series, 45 patients (primary, *n* = 11; referred, *n* = 34) were treated with multiple irrigation and debridement. Cefazolin was used as empiric antibiotics, then changed according to microbiologic study. Previously used antibiotics, treatment delay, surgical history of the referred patients were reviewed. Identified microorganisms, required surgical intervention, hospital stay, radiologic outcome, functional outcomes were compared between both groups.

**Results:**

In the referred patients, methicillin-resistant *Staphylococcus aureus* (MRSA) was commonly found and cefazolin was susceptible in only 15% of the cases. Longer hospital stay, prolonged antibiotic therapy, more surgical intervention including flap surgery was required to treat the referred patients. Postoperative pain was not severe in daily activities, but the final range of motion was significantly less in the referred patients compared to the primary patients.

**Conclusions:**

This study suggests that in the treatment of uncontrolled septic arthritis of the digits, antibiotic agents covering MRSA may shorten the duration of antibiotic therapy in areas of high MRSA incidence. Besides, more number of I & D including flap surgery may be required for the referred patients compared with the primary patients. These findings can help the surgeon in setting up a treatment plan or in counseling of referred patients with uncontrolled septic arthritis of the digits.

## Background

Septic arthritis of the digits is an urgent condition that requires prompt surgical debridement and appropriate antibiotic therapy. However, not all patients can be treated quickly. Patients often visit the hospital late, the diagnosis may be delayed, or initial treatment can be insufficient. Delayed treatment can lead to irreversible cartilage damage, soft tissue destruction, or osteomyelitis, limited range of motion (ROM), joint arthrodesis, and amputation [1–3]. Besides, it is difficult to treat the delayed septic arthritis with simple irrigation and debridement (I & D), and often patients are referred to the regional tertiary hospital for an uncontrolled infection. Several guidelines for antibiotic therapy for septic arthritis classified the patients according to the risk factors [4, 5]. Traditionally, narrow-spectrum antibiotic agents (flucloxacillin, cefazolin) covering methicillin-sensitive *Staphylococcus aureus* (MSSA) and *Streptococcus* species has been advocated as an empiric antibiotic therapy for the patients without a risk factor [3, 4, 6, 7]. Some recent reports recommended agents against methicillin-resistant *Staphylococcus aureus* (MRSA) as empiric treatment for the patients without risks [5, 8]. However, the choice of empiric antibiotic agents for the referred patients is not well studied. In addition, the frequency and type of surgical procedures required to treat the referred patients are rarely described.

In this study, we reviewed the treatment history of the referred patients, and compared the microorganisms and clinical course of the primary and referred patients, to identify the appropriate empiric antibiotic agents and surgical treatments required for the referred patients. We hypothesized that narrow-spectrum antibiotics against MSSA and *Streptococcus* species were insufficient for the referred patients, and that I & D and other procedures were more frequently required in the referred patients than in the primary patients.

## Methods

After obtaining approval of the study protocol from the Institutional Review Board (IRB number 05–2020-007; IRB waived the need for informed consent), we retrospectively reviewed consecutive patients (*n* = 64) with presumptive chronic septic arthritis of the digits who were treated primarily in our hospital or referred to our hospital due to uncontrolled infection between January 2010 and December 2017. One surgeon (the first author) who was level 3 (specialist-experienced) according to Tang’s levels of surgical expertise performed all surgeries [9]. The definite diagnosis of septic arthritis of the digits was made after the surgery as having at least one of the following criteria: (1) growth of microorganisms from samples obtained during the operation, (2) intraoperative pus in the joint, and (3) neutrophils in the microscopic analysis of samples obtained during surgery (neutrophils ≥10 in any of the 5 high power (× 40) fields) [2, 10–13]. We excluded patients who did not meet any of the criteria of septic arthritis (*n* = 11), patients diagnosed with tuberculous arthritis (*n* = 2), patients diagnosed with rheumatoid arthritis (*n* = 4), and patients with metal implants (*n* = 2). Finally, we included 11 patients treated primarily and 34 patients who were referred to our hospital after initial treatment. Twenty-seven distal interphalangeal joints, 15 proximal interphalangeal joints, 2 thumb interphalangeal joints, and 1 thumb metacarpophalangeal joint were involved. The mean follow-up period was 16 months (range, 11–36 months).

**Fig. 1:**
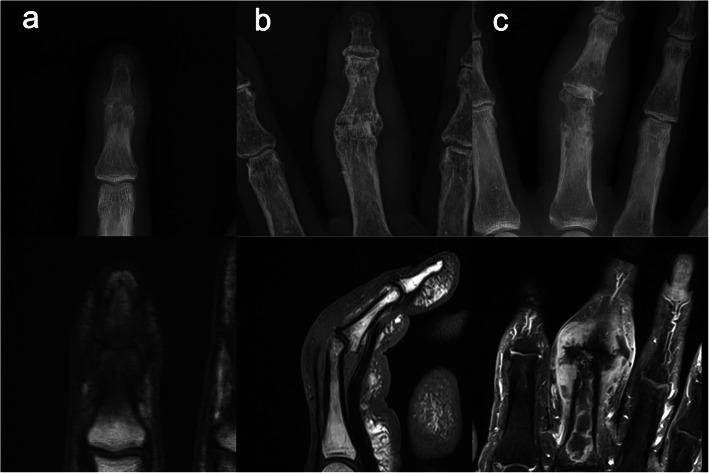
**a** The right index finger of a 35-year-old man. The distal interphalangeal joint shows joint space narrowing in a simple radiograph, while T1 weighted image shows intramedullary marked low signal intensity, indicating osteomyelitis. **b** The right long finger of an 80-year-old woman. The proximal interphalangeal joint shows joint space narrowing and radial angulation deformity in a simple radiograph; however, a normal marrow signal is shown in the T1 weighted image. **c** The right long finger of a 58-year-old woman. The proximal interphalangeal joint shows definite osteolysis in a simple radiograph. The proximal phalanx reveals extensive intraosseous abscess in contrast-enhanced T1 weighted image

Preoperatively, we obtained information including surgical debridement and empiric antibiotic therapy in the referred patients based on the record of the previous hospital. Both groups underwent history taking of symptom onset, presence of penetrating event, medications associated with systemic immunosuppression (corticosteroid, methotrexate, biologics) [14], immune compromising comorbidities such as diabetes mellitus and chronic kidney disease [15, 16], and risk factors for atypical organism infections (precedent cellulitis, sexual activity, elderly patients with urinary tract infections, intravenous drug abuse, working as a gardener, rheumatoid arthritis, and animal bites) [5]. All patients underwent magnetic resonance imaging (MRI) in addition to simple radiography to detect the possibility of combined osteomyelitis and unexpected intraosseous abscess in a three-dimensional plane. Combined osteomyelitis was diagnosed if any evidence of definite bone destruction, intramedullary confluent marked low signal intensity, or bone marrow edema with soft tissue ulcer was observed in T1 weighted image (Fig. 1) [17].

### Surgical techniques

For interphalangeal joints of the 2nd-5th digits, we applied a finger tourniquet to the digit and excised the skin and soft tissue around a drainage sinus until fresh bleeding. If the skin hole was not sufficient to open the joint capsule or there was no drainage sinus, an additional midaxial approach was made [2]. Debridement of the intraarticular surface and curettage of the devitalized osteocartilaginous fragments were performed through the excised skin opening or the midaxial incision. Nonviable extensor tendon was excised. Intraosseous abscess identified on MRI was treated by curettage through the joint or making additional cortical windows. The wound was left open with a bulky dressing.

For the thumb joints, a volar or a dorsolateral approach was made according to the site of drainage sinus. For the metacarpophalangeal joint, the dorsoulnar incision was made and the joint was opened between the ulnar collateral ligament and the extensor pollicis longus. For the interphalangeal joints, the A2 pulley was cut, and the joint was explored through the volar plate. Incision of the oblique pulley was not attempted to prevent the bowstring effect of the flexor pollicis longus. After debridement of the skin and soft tissue around the drainage sinus, the joint was opened and debrided. The wound was left open with a bulky dressing.

### Postoperative care and additional procedures

Dressing change for the open wound was performed daily. If purulent discharge continued, repeated debridement and irrigation with normal saline were performed every 2–3 days until the discharge stopped. Antiseptics, such as octenidine or polyhexanide, were not used to prevent cartilage damage [18, 19]. Soaking or wet dressing was not used. If the soft tissue defect was not healed by the intention of the surrounding tissue, flap surgery was performed to cover the defect.

Empirically, patients without risk factors for atypical organisms received intravenous cefazolin (primary patients, *n* = 10; referred patients, *n* = 32). In three cases of animal bite injury (primary patient, *n* = 1; referred patients, *n* = 2), they received intravenous amoxicillin to cover the *Pasteurella* species infection [4, 5]. Antibiotics were changed according to the gram stain result, identified microorganism, and susceptibility test. Effective antibiotics were continued for at least 4 weeks after the discharge stopped [20]. If the wound improved and oral form of antibiotics was available, the patient was discharged with oral antibiotics. If not, the patient received intravenous antibiotics continuously. Complete blood cell count, liver panel, and renal panel were checked twice a week. In case of adverse effect, the infectious disease specialist decided whether the causative antibiotic agent should be ceased or changed.

**Fig. 2:**
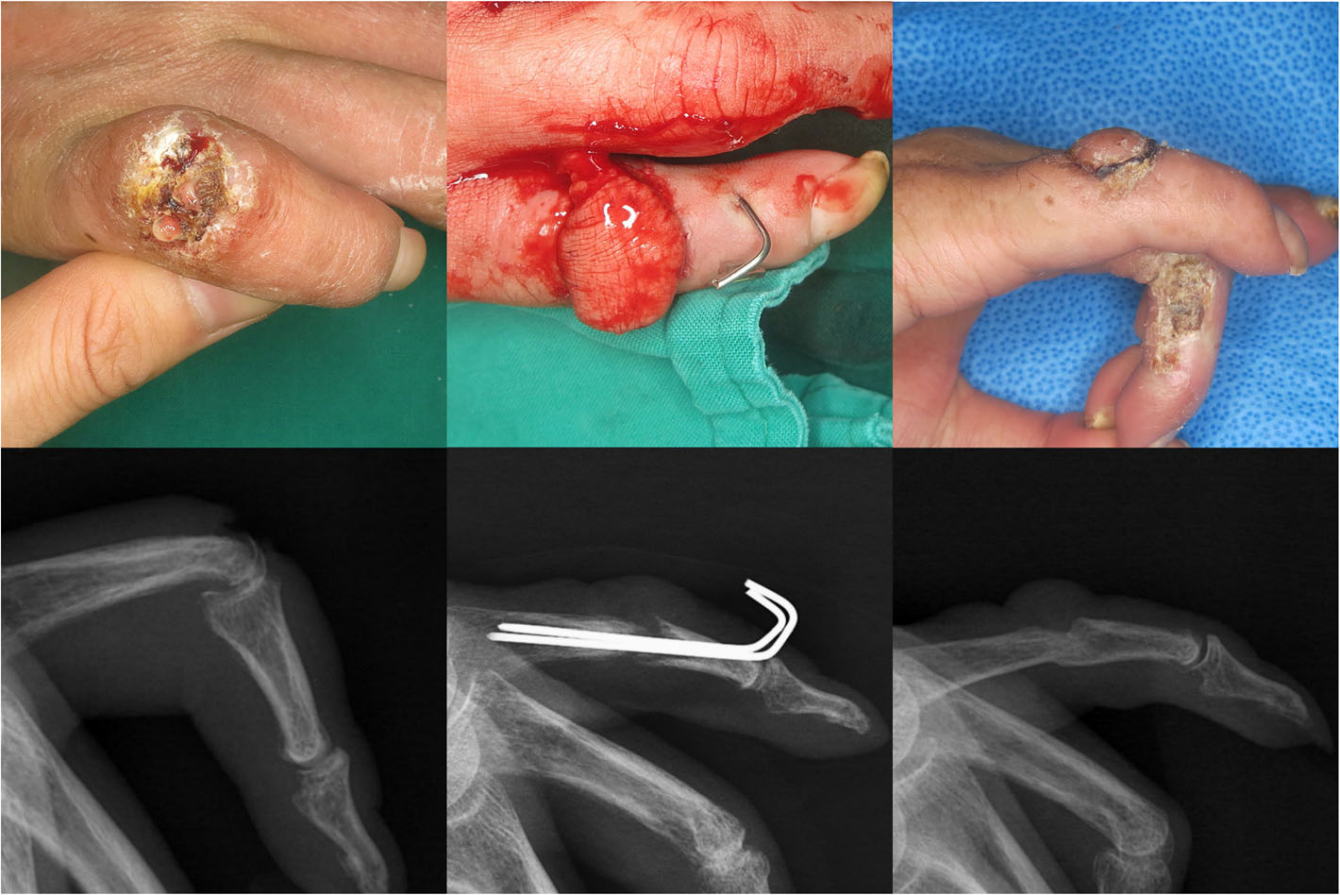
The right hand of a 53-year-old man, referred due to uncontrolled infection. The proximal interphalangeal joint of the little finger shows a chronic wound with discharge. After several debridement and irrigation, the soft tissue defect is covered using a heterodigital island flap with temporary Kirschner wire fixation. The joint united spontaneously

If additional flap surgery was performed, bone fixation with immobilization was applied to protect the flap position for 2 weeks (Fig. 2). Otherwise, active finger ROM exercise inside the bulky dressing was allowed to prevent stiffness.

### Clinical data and outcome evaluation

We recorded active ROM of the involved joint and Quick Disabilities of the Arm, Shoulder, and Hand (DASH) score and checked the simple radiographs at 3, 6, and 12 months postoperatively, and annually thereafter. We reviewed the data on the site of involvement, combined osteomyelitis, hospital stay, antibiotic therapy, microorganisms, and surgical intervention in the medical record.

### Statistical analysis

We performed the Shapiro-Wilk test for normality. According to the result of the Shapiro-Wilk test, Student’s *t*-test was used for independent parametric continuous variables and the Mann-Whitney *U*-test was used for independent non-parametric continuous variables. The Fisher’s exact and chi-squared tests were used to compare discrete variables. Statistical significance was set at *p* < 0.05.

## Results

Table 1 shows the patient demographics and the inclusion criteria. The referred patients were significantly older and had longer duration of delay from symptom onset than the primary patients. All primary patients underwent surgical drainage, whereas only 8 referred patients (23%) underwent surgical drainage at the previous hospital. In the referred group, 5 patients without penetration event were misdiagnosed with osteoarthritis at the first visit to the previous hospital. In addition, 13 iatrogenic penetration events were related to the treatment procedure of osteoarthritis such as steroid injection (*n* = 6), acupuncture (*n* = 5), and rupture of a mucous cyst (*n* = 2).

**Table 1:** Demographics of the patients and inclusion criteria

			Primary patients (*n* = 11)	Referred patients (*n* = 34)	*p*-value
Age^a^ (year)			48.5 (3.47)	60.5 (4.02)	**0.001**
Sex (male/female)			4/7	18/16	0.491
Immunocompetent disease or immunosuppressive drug (yes/no)			7/4	20/14	1.0
DIP/PIP/Thumb			8/3/0	19/12/3	0.120
Penetration event (traumatic/iatrogenic/no)			9/0/2	16/13/5	**0.048**
Specific clinical history			1 (animal bite)	2 (animal bite)	1.0
Delay from symptom onset to treatment in the first visiting hospital^b^ (day)			9 (7–14) [2–21]	21 (7–28) [3–70]	**0.042**
Duration of empiric antibiotics in the previous hospital^c^ (day)			–	7.5 (5–10.25) [3–28]	**–**
Delay from symptom onset to treatment in the present hospital^c^ (day)			–	38 (21–91) [13–168]	**–**
Surgical drainage at the first visiting hospital (yes/no)			11/0	8/26	**< 0.001**
Culture (+)	Pus (+)	Neutrophils ≥10/HPF	2	17	**–**
		Neutrophils < 10/HPF	5	7	
	Pus (−)	Neutrophils ≥10/HPF	–	2	
		Neutrophils < 10/HPF	3	1	
Culture (−)	Pus (+)	Neutrophils ≥10/HPF	–	5	
		Neutrophils < 10/HPF	1	2	
	Pus (−)	Neutrophils ≥10/HPF	–	–	

Significant values shown in bold

*DIP* distal interphalangeal, *PIP* proximal interphalangeal, *HPF* high power field

^a^Student’s t-test. Mean (standard deviation) presented

^b^Mann-Whitney *U*-test. Median (25th–75th percentiles) [range] presented

^c^Non-parametric distribution. Median (25th–75th percentiles) [range] presented

Overall, 45 microorganisms were detected in 37 patients, with *S. aureus* (51.1%) being the most common microorganism, followed by *Streptococcus* species (11.1%). The primary and referred patients had MSSA and MRSA, respectively, as the most common microorganism. Among the patients with identified microorganisms, cefazolin was susceptible in 90 and 15% of the primary and referred patients, respectively (Table 2). Cefazolin in 24 referred patients were changed to cover MRSA (*n* = 14) or other organisms (*n* = 10) at 5 ~ 8 days after the admission. Before the referral, 29 patients received one empiric antibiotic agent and 5 patients received two agents. The third-generation cephalosporin was the most commonly used agent (33%) followed by oral amoxicillin-clavulanate (18%), and 20 oral and 19 intravenous agents were prescribed. Among the 39 antibiotics, only 6 agents were effective in the identified microorganisms (Table 3). To sum up, only 2 referred patients received both I & D and susceptible antibiotics.

**Table 2:** Microbiological results

		Primary patients	Referred patients	*p*-value
Culture-positive/Total patients		10/11	27/34	0.657
Mono/Polymicrobial		10/0	20/7	0.155
Identified Organisms		10	35	
Gram-positive cocci	MSSA	8	1	**< 0.001**
	MRSA	0	14	**0.006**
	*Streptococcus* species	0	5	0.295
	*Enterococcus faecalis*	0	1	1.0
Gram-negative bacteria	*Pseudomonas aeruginosa*	1	3	1.0
	*Pasteurella* species	1	2	1.0
	*Eschericia coli*	0	2	1.0
	*Enterobacter cloacae*	0	2	1.0
	*Proteus vulgaris*	0	2	1.0
	*Prevotella* species	0	1	1.0
	*Aeromonas sobria*	0	1	1.0
Fungal infection	*Candida parapsilosis*	0	1	1.0
Susceptibility to cefazolin (yes/no)		9/1	4/23	**< 0.001**

Significant values shown in bold

*MRSA* methicillin-resistant *Staphylococcus aureus*, *MSSA* methicillin-sensitive *Staphylococcus aureus*

**Table 3:** Empiric antimicrobial agents of the referred patients in the previous hospital

Classification		Antimicrobial agent	Total number of administration	Susceptible to the identified microorganism / Cases with no growth of microorganism
Penams	Penicillin	IV nafcillin	1	0/0
	Beta lactam/Beta lactamase inhibitor	Oral amoxicillin-clavulanate	7	1 (MSSA) /2
Cephems	1st generation cephalosporin	IV cefazolin	5	0/3
		Oral cephalexin	1	0/0
	2nd generation cephalosporin	IV cefotetan	2	0/0
		IV cefotiam	1	1 (*Streptococcus* specis)/0
	3rd generation cephalosporin	IV cefotaxime	4	1 (*Pasteurella multocida*) /0
		IV ceftriaxone	5	1 (*Enterobacter cloacae*)/0
		Oral cefpodoxime	4	2 (*Streptococcus* species, *Eschericia coli*)/1
Aminoglycoside		IV netilmicin	1	0/0
Lincosamides		Oral clindamycin	3	0/0
		Unidentified oral form	5	Not available/1

*IV* intravenous, *MSSA* methicillin-sensitive *Staphylococcus aureus*

**Fig. 3:**
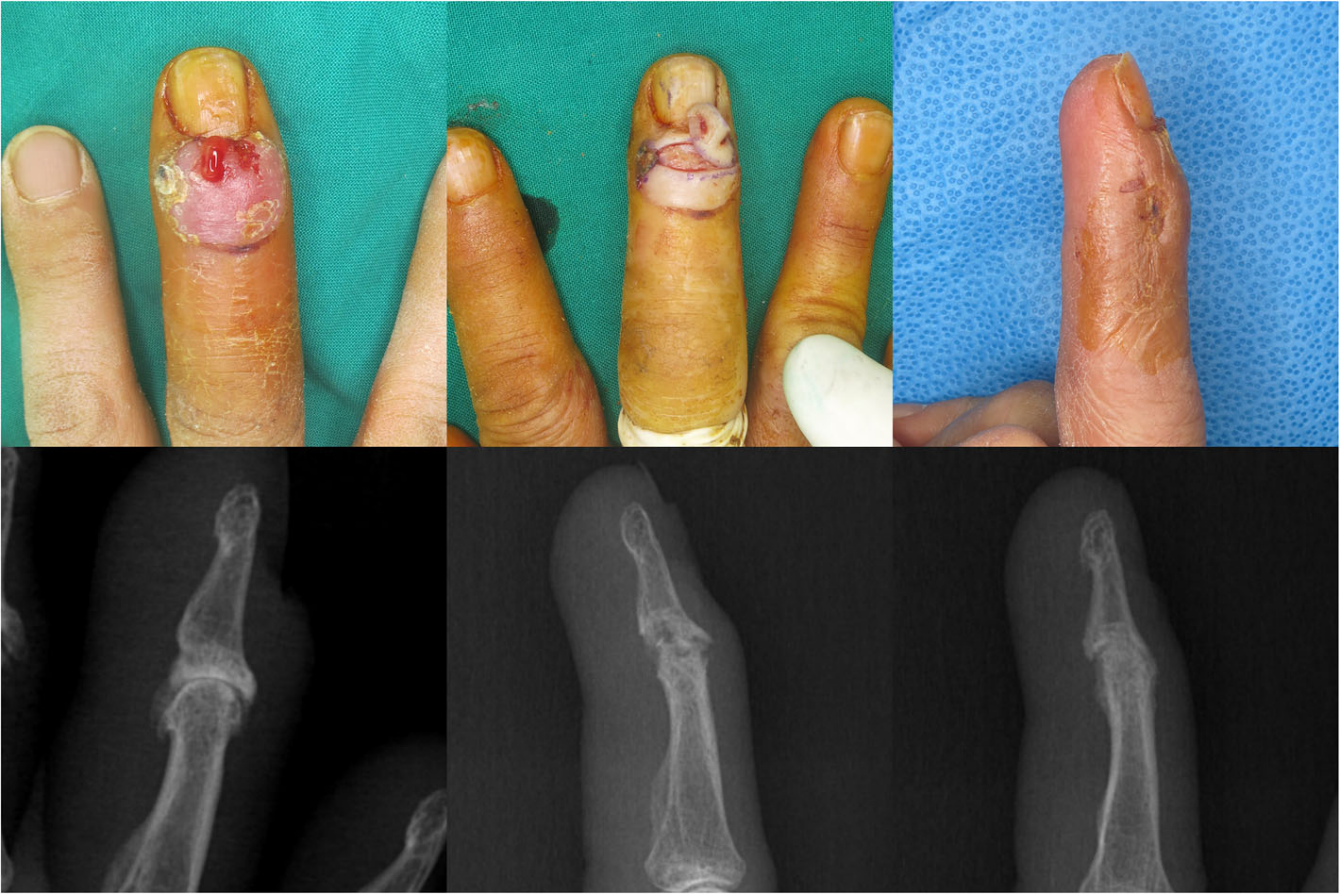
The right hand of a 62-year-old woman, treated primarily. The distal interphalangeal joint of the long finger shows a drainage sinus. The skin around drainage sinus is excised and joint debridement is performed through additional midaxial incision. The joint united spontaneously

**Fig. 4:**
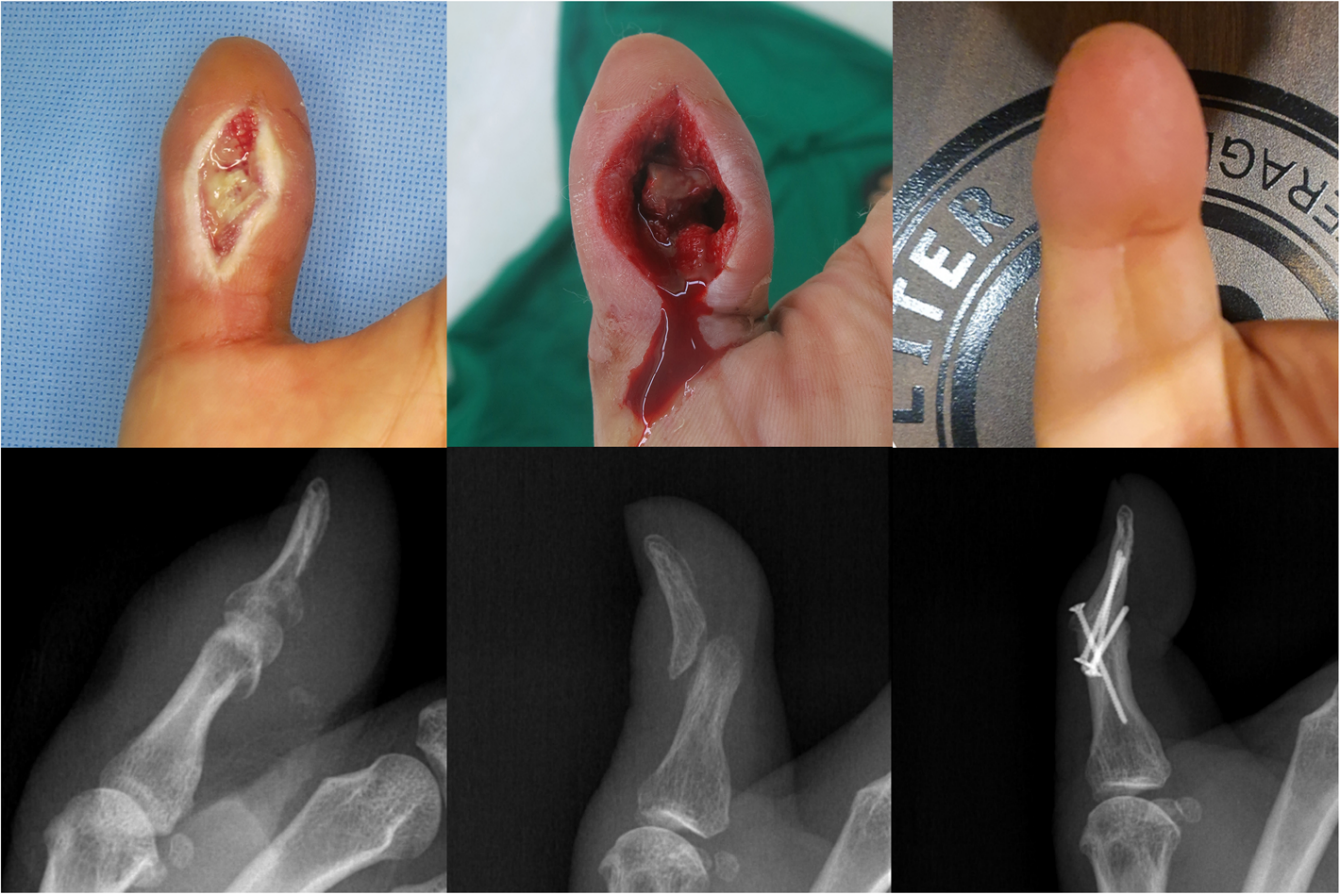
The left hand of a 51-year-old man, referred due to uncontrolled infection. The interphalangeal joint of the thumb is debrided through a volar incision and additional fixation is performed after the infection controlled. Bone union is achieved at the last visit

All patients were successfully treated without amputation or intended arthrodesis. The referred patients required significantly more frequent I & D, prolonged antibiotic treatment, and longer hospital stay. Although the *p*-value was not < 0.05, only the referred patients required flap surgery. In two primary patients, antibiotic therapy was stopped after 3 weeks due to adverse reactions. Of all the joints, 44.1% of the referred patients and 9.1% of the primary patients united spontaneously, and the final ROM of the referred patients was significantly lower than that of the primary patients (Fig. 3). Forty-three and two patients chose ‘no difficulty’ and ‘mild difficulty’ in pain items of Quick DASH, resulting in an insignificant difference between both patients (Table 4). No case showed a relapsed infection, but one patient underwent additional fixation due to instability (Fig. 4).

**Table 4:** Accompanied osteomyelitis, clinical course, and results of the patients

	Primary patients (*n* = 11)	Referred patients (*n* = 34)	*p*-value
Accompanied osteomyelitis (yes/no)	6/5	28/6	0.062
Number of incision and debridement^a^	2 (1–2) [1–3]	4 (3–6) [1–10]	**< 0.001**
Flap surgery (yes/no)	0/11	7/27	0.168
Number of microorganisms^a^	1 (1–1) [0–1]	1 (1–1) [0–3]	0.725
Duration of antibiotic treatment^a^ (wk)	4 (4–6) [3–6]	6 (6–6) [5–7]	**< 0.001**
Hospital stay^a^ (day)	25 (23–29) [22–40]	33 (28–37) [21–45]	**0.005**
Spontaneous union (united/not united)	1/10	15/19	0.067
Range of motion at last follow-up^a^ (°)	20 (10–25) [0–30]	5 (0–10) [0–20]	**< 0.001**
Quick DASH score^a^	11.3 (5–18.2) [5–18.2]	15.0 (9.1–15.0) [5–38.6]	0.121

Significant values shown in bold

*DASH* Disabilities of the Arm, Shoulder, and Hand

^a^Mann-Whitney *U*-test. Median (25th–75th percentiles) [range] presented

## Discussion

After reviewing patients with septic arthritis of the digit, we observed that diagnostic delay and insufficient surgical drainage were more common in the referred patients than in the primary patients. In the referred patients, MRSA was the most common pathogen, and most of the previously used antibiotics were not effective against the organisms identified at our hospital. The referred patients required more surgical intervention including flap surgery, prolonged duration of antibiotic therapy, and longer hospital stay than the primary patients.

Although septic arthritis is generally considered as a surgical emergency [2, 21], treatments are often delayed when it affects the digits [1, 3]. In addition, diagnosis is difficult in patients with native joints, and can be further impeded due to postoperative pain, particularly in patients after finger surgery [3]. In the present study, 18 (53%) of the referred patients were initially diagnosed with osteoarthritis at the previous hospital. Discerning the early stage of septic arthritis from noninfectious conditions might be difficult, especially considering the high incidence of osteoarthritis in the elderly. Moreover, even after the physician suspected an infectious condition, most cases were regarded as superficial wound infection, cellulitis, and early fingertip infections, except the 8 cases with surgical drainage [21]. Thus, surgical treatment might have been delayed or not performed owing to an inaccurate diagnosis, and joint destruction was likely to progress. Since antibiotics are not effective against necrosis, treatment failure was likely to have occurred owing to the lack of effective early surgical debridement. Thus, we believe that suspecting septic arthritis is an important indicator for early surgical treatment with antibiotic therapy when old patients present with swollen joints of the digit.

In septic arthritis of the native finger joints, *S. aureus* was the most frequent isolated microorganism, followed by *Streptococcal* species; further, MRSA was detected in lesser number of cases than was MSSA [1, 7, 10, 21, 22]. Thus, conventional narrow-spectrum antibiotics such as penams and the first-generation cephalosporins are recommended in primary cases. However, only 14 referred patients in this study were treated with conventional narrow-spectrum antibiotics during primary treatment. While the second- or third-generation cephalosporins were indicated for those with an allergy to penicillin or those at the risk of atypical organism infections [5], of the referred patients only two reported a history of animal bites and none reported penicillin allergies. Considering that 21 g-positive cocci were confirmed among the 35 microorganisms identified in the referred patients, this inappropriate use of second- or third-generation cephalosporins might be another cause of treatment failure. Conventional narrow-spectrum antibiotics appeared to be less effective in the referred patients than in the primary patients. Cefazolin was changed to other agents in 24 referred patients and this delay in the use of effective antibiotics led to extended durations of antibiotic therapy and hospital stay. Considering the high incidence of MRSA in the referred patients, we believe that physicians should suspect MRSA as a causative microorganism if gram-positive cocci are identified. Although the conventional narrow-spectrum antibiotics had several advantages [23], agents covering MRSA from the beginning of the treatment period might have shortened the duration of therapy for the referred patients.

In cases combined with osteomyelitis, we partially or completely debrided the cartilage. As more cartilage and subchondral debridement were performed in the referred patients, more of these patients showed irreversible osteochondral damage than the primary patients. Additionally, referred patients often showed soft tissue defects that could not be rectified by secondary healing. To address osteochondral damage and soft tissue defects, previous studies have recommended intended arthrodesis with shortening as an additional procedure [1, 3]. In our cases, 20.5% of the referred patients required additional procedure and we performed flap surgery instead of intended arthrodesis, to avoid infection after internal fixation. Although we did not perform arthrodesis, some joints were spontaneously united, and there was only one complaint of instability. Thus, we believe that this additional procedure should be considered as a feasible option especially for the referred patients, and flap surgery might be used instead of arthrodesis.

Whereas several previous reports have focused on primary patients, our study presents data on patients referred due to uncontrolled infection. Furthermore, all patients in this study underwent operations from a single surgeon in a tertiary clinic; thus, consistency in the surgical techniques and antimicrobial protocols were assured. Although this study has some strengths, it also has weaknesses. First, as the finger joints are small, samples obtained during surgery might often be insufficient for the culture study. This caused false-negative results, especially in the primary patients, and eventually lowered the number of primary patients included. Second, the neutrophil counts obtained in histologic study are not an established criterion for septic arthritis. Neutrophil (CD 15+ cell) counts in synovial tissue should only be used as a supplementary tool for diagnosing septic arthritis [24]. Moreover, the optimal cutoff value of synovial neutrophil count has not been well-studied in septic arthritis. Thus, we used the cutoff value used for identifying septic total arthroplasty [11] since neutrophil counts are reportedly similar in septic arthritis and septic total arthroplasty [12]. Third, both groups were not similar in terms of age and delay from symptom onset. In the referred group, both older age and longer treatment delay might also affect the hospital course negatively. Fourth, suboptimal dosage or insufficient surgical drainage was not considered as inappropriate treatments. Thus, the number of effective treatments might be counted higher than the real number. Fifth, our result could not give a clear guideline about the required number of surgical procedures and the duration of antibiotics use. Surgeons can choose either primary extensive resection or sequential gradual resection as long as the debridement extends beyond the necrotic tissues. Besides, the duration of antibiotics use could be shortened according to recent studies [10, 25]. Thus, future studies, including large cohorts (to match age and treatment delay) and focusing on the extent of surgical debridement and shorter duration of antibiotics use are warranted.

## Conclusion

Diagnostic delay and insufficient surgical drainage might be associated with treatment failure, and our results suggest that the more time elapsed before adequate local therapy was performed, the less the effect of antibiotic administration. For patients referred due to treatment failure, antibiotics were more frequently changed to cover MRSA and more numbers of surgical debridement, including flap surgery, were performed compared with the primary patients. Besides, prolonged duration of antibiotic therapy and longer hospital stay were required in the referred patients than in the primary patients. These findings can help surgeons in setting up a treatment plan or in counseling referred patients with uncontrolled septic arthritis of the digits.

## Data Availability

The datasets generated during and analyzed during the current study are available from the corresponding author on reasonable request.

## Abbreviations

ROM: Range of motion
I & D: Irrigation and debridement;
MSSA: Methicillin-sensitive *Staphylococcus aureus*
MRSA: Methicillin-resistant *Staphylococcus aureus*
MRI: Magnetic resonance imaging
DASH: Disabilities of the Arm, Shoulder, and Hand
